# Quantifying Risk Pathway Crosstalk Mediated by miRNA to Screen Precision drugs for Breast Cancer Patients

**DOI:** 10.3390/genes10090657

**Published:** 2019-08-28

**Authors:** Yingqi Xu, Shuting Lin, Hongying Zhao, Jingwen Wang, Chunlong Zhang, Qun Dong, Congxue Hu, Desi Shang, Li Wang, Yanjun Xu

**Affiliations:** College of Bioinformatics Science and Technology, Harbin Medical University, Harbin 150081, China

**Keywords:** breast cancer subtype, miRNA, pathway, crosstalk network, precision drugs

## Abstract

Breast cancer has become the most common cancer that leads to women’s death. Breast cancer is a complex, highly heterogeneous disease classified into various subtypes based on histological features, which determines the therapeutic options. System identification of effective drugs for each subtype remains challenging. In this work, we present a computational network biology approach to screen precision drugs for different breast cancer subtypes by considering the impact intensity of candidate drugs on the pathway crosstalk mediated by miRNAs. Firstly, we constructed and analyzed the subtype-specific risk pathway crosstalk networks mediated by miRNAs. Then, we evaluated 36 Food and Drug Administration (FDA)-approved anticancer drugs by quantifying their effects on these subtype-specific pathway crosstalk networks and combining with survival analysis. Finally, some first-line treatments of breast cancer, such as Paclitaxel and Vincristine, were optimized for each subtype. In particular, we performed precision screening of subtype-specific therapeutic drugs and also confirmed some novel drugs suitable for breast cancer treatment. For example, Sorafenib was applicable for the basal subtype treatment, Irinotecan was optimum for Her2 subtype treatment, Vemurafenib was suitable for the LumA subtype treatment, and Vorinostat could apply to LumB subtype treatment. In addition, the mechanism of these optimal therapeutic drugs in each subtype of breast cancer was further dissected. In summary, our study offers an effective way to screen precision drugs for various breast cancer subtype treatments. We also dissected the mechanism of optimal therapeutic drugs, which may provide novel insight into the precise treatment of cancer and promote researches on the mechanisms of action of drugs.

## 1. Introduction 

Breast cancer is the most common cancer type that leads to women’s death, especially in China. The high heterogeneity of breast cancer makes it a great challenge to adopt therapeutic options [1], because a heterogeneous group of diseases may exhibit distinct features in terms of histological, prognostic, and clinical outcomes [2]. At present, breast cancer can mainly be classified into four primary subtypes, including her2-enriched, luminal A, luminal B, and basal-like [3,4], distinguished by the expression of some signature genes such as the estrogen receptor (ER), progesterone receptor (PR), and HER2. Different subtypes have distinct biological behaviors and prognosis, and also exhibit various responses to drug therapy [5,6]. Thus, further research on the biological heterogeneity of each subtype of breast cancer will be an effective way to improve the therapeutic efficacy and prognosis of breast cancer [7].

The oncogenesis processes may result from the dysregulations of a series of important biological pathways [8]. Some studies have shown that the pathway crosstalk exists extensively in the processes of development and cell fate [9,10,11]. Cancer cells have been found to be able to establish alternative signaling pathways through crosstalk to adapt to drug treatment. In addition, crosstalk can also promote cancer therapy by inhibiting the main oncogenic pathways. The inhibition of functional redundancy and pathway crosstalk that promotes the survival of cancer cells can prevent the resistance in tumor treatment [12]. Therefore, it is essential to dissect the crosstalk of dysfunctional pathways and further capture the key molecules that mediate this functional crosstalk in breast cancer.

MicroRNAs are endogenous, non-coding RNA molecules that have been widely regarded as important post-transcriptional regulators by damping the expression level of their target genes. In recent years, studies have indicated that miRNAs are important component elements of biological pathways [13]. They regulate the function of biological pathways through target genes, and then work together with them to disrupt the pathways of diseases. According to estimates, many microRNAs play vital roles by regulating processes that are implicated with the development of cancer [14], such as proliferation, apoptosis, cell cycle, angiogenesis, etc. Some studies suggest that the crosstalk between miRNAs and the Wnt pathway may impact oncogenesis, cancer metastasis, and even drug-resistance processes [15]. Furthermore, miRNAs can also mediate the functional crosstalk of pathways related with oncogenic processes by targeting their shared or interacted genes, thus promoting the initiation and progression of tumors. 

In recent years, miRNAs have shown great promise to serve as a target for drug therapy of cancer. More importantly, some studies have nominated miRNA-based therapy as a promising strategy for the treatment of breast cancer [16]. Some evidence demonstrates that drugs could modulate the expression of miRNAs in various diseases as well. For example, an experiment has validated that simvastatin could lead to cell death of breast cancer by up-regulating miR-140-5p [17]. Triiodothyronine has been demonstrated to modulate miR-204 and thus facilitate the proliferation process in breast cancer [18]. Especially, Shenoda et al. have also demonstrated that miRNA could mediate the expression of genes related with drug metabolism [19]. Furthermore, Liu et al. have established a database SM2miR [20], which provides a comprehensive resource about the influences of drugs on miRNA expression and offers unprecedented opportunities for researchers on the screening and action mechanism of drugs for disease treatment. In addition, our previous research also displays that miRNA participates in the crosstalk among pathways that play important roles in cancer development [21], indicating that it might be more effective for screening cancer treatment to evaluate the effects of drugs on the miRNA-mediated crosstalk between pathways.

**Figure 1:**
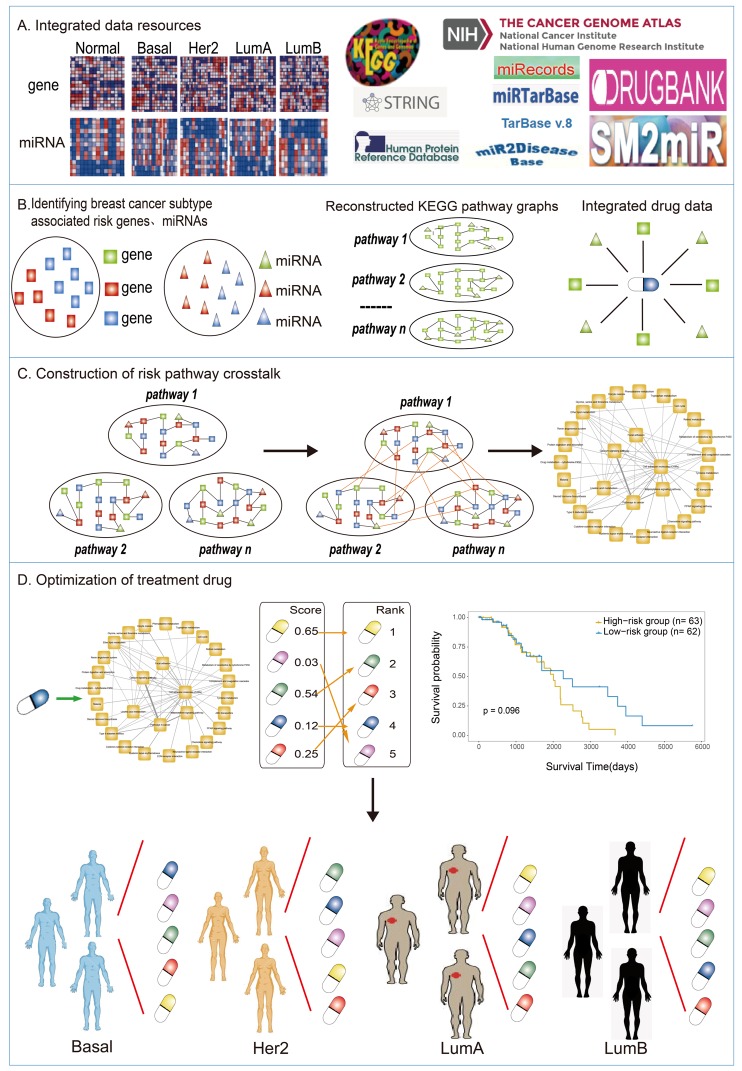
The workflow of optimizing drugs for different subtypes of breast cancer. (**A**) In this work, breast cancer was taken as the research model. Firstly, we integrated related data resources, including gene/miRNA expression profile of each breast cancer subtype and matching patients’ survival information, miRNA-target relationship data, PPI network and pathway data, and drug and drug target data. (**B**) We identified the differential genes/miRNAs of each breast cancer subtype, and then reconstructed KEGG pathway based on miRNA-target interactions, which contained both genes and miRNAs. We also screened the target genes and target miRNAs of Food and Drug Administration (FDA)-approved anticancer drugs. (**C**) Identification of breast cancer subtype-associated risk pathways based on the differential genes/miRNAs, and calculated crosstalk for any two interrelated risk pathways. Furthermore we constructed miRNA-mediated specific pathway crosstalk networks in different subtypes of breast cancer, respectively. (**D**) The effectiveness assessment of drugs on dysfunction crosstalk network to screen candidate drugs, combined with survival analysis to optimize drugs for each breast cancer subtype. (See Methods section for details.)

In order to match the best treatment for breast cancer, in the present study, we firstly integrated the disease high-throughput molecular profiles, miRNA regulation data, and pathway and drug data to construct and analyze the miRNA-mediated pathway crosstalk network for various breast cancer subtypes. Then, we derived a novel computational method to screen precision drugs for different breast cancer subtypes by quantifying the impact intensity of candidate drugs on the pathway crosstalk mediated by miRNAs. Finally, survival analysis was combined for further screening and optimization of the drugs for breast cancer treatment (Figure 1). In summary, our study proposes an effective method to screen precision drugs for various breast cancer subtype treatments. We also dissected the mechanism of optimal therapeutic drugs, which may promote the shift from inexact medicine to precision life science.

## 2. Material and Methods

### 2.1. Sample Matched miRNA/Gene Expression Profiles and Clinical Data

The matched miRNA and gene expression data of breast cancer were downloaded from TCGA (The Cancer Genome Atlas) database (http://tcga-data.nci.nih.gov/), including 553 human breast cancer samples and 87 normal samples. These breast cancer samples were divided into four subtypes, including basal-like (n= 97), Her2 (n = 47), luminal A (n = 291) and luminal B (n = 118) according to the guidelines in Cirielloet et al. [22]. All selected expression datasets were log2-transformed, then standardized. Furthermore, clinical survival data of these samples in each subtype were also obtained.

#### 2.1.1. miRNA-Target Relationship Data

In this study, we collected experimentally verified miRNA-target interactions data from four well-known data resources: miRTarBase [23], mir2Disease [24], miRecords (V4.0) [25], and TarBase (V6.0) [26]. MiRNA-target relationships in homo species were extracted and combined together to obtain a more comprehensive dataset. In total, 57,863 miRNA-target relationships involving 579 miRNAs and 14,652 target genes were collected and used for further analysis.

#### 2.1.2. PPI Network and Pathway Data

The protein–protein interaction (PPI) network data used in this study were integrated from two databases, HPRD (Human Protein Reference Database) and STRING (Search Tool for the Retrieval of Interacting Genes/Proteins) [27,28]. The interactions stored in HPRD were mainly from experimental validation and text mining. For each recorded entry in the STRING database, a weighted score was given to measure their confidence of interaction by considering multiple factors. To collect high-quality interaction data, we only extracted interactions with a confidence score ≥900. Then, we combined interactions from the HPRD and STRING databases. The pathway data used in this study for functional analysis were obtained from the KEGG (Kyoto Encyclopedia of Genes and Genomes) database [29].

#### 2.1.3. Drug and Drug Target Data

In this study, according to our research purpose, in order to improve the practicability of our study, the candidate drugs need to satisfy two requirements simultaneously. Firstly, existing gene targets and regulatory effects on miRNA have to be confirmed. Secondly, the drugs have to have been approved by US Food and Drug Administration (FDA, https://www.fda.gov/), which are prescribed for cancer treatment. We extracted drugs and drug targets from DrugBank [30] and SM2miR [20]. Finally, a total of 36 anticancer drugs were used in this study. The complete information of the 36 anticancer drugs can be found in Supplementary Table S1, including drug ID and drug targets.

### 2.2. Reconstructed KEGG Pathway Graphs 

The reconstructed KEGG (Kyoto Encyclopedia of Genes and Genomes) pathway graphs contained both genes and miRNAs, replicating real biological pathways. We firstly collected 220 KEGG pathway data and converted them into undirected graphs with genes as nodes and their interactions as edges by using our previously developed R package “iSubpathway Miner” [31]. Then, we reconstructed these pathways by wiring miRNAs into these pathways through integrating miRNA-target relations and pathway data. More details, if target genes of a specific miRNA were over-represented within a pathway, the miRNA was wired into the pathway by connecting with target genes within the pathway. The hypergeometric test was used to evaluate the significance of enrichment. The formulas is as follows:

$$P=1-\overset{q-1}{\underset{t=0}{\sum }}\frac{\left(\begin{array}{c} l\\ t \end{array}\right)\left(\begin{array}{c} n-l\\ m-t \end{array}\right)}{\left(\begin{array}{c} n\\ m \end{array}\right)}$$

where n represents the number of background genes (all genome-wide genes), m is the number of genes involved in a given pathway, l is the number of target genes for a specific miRNA, and q is the number of miRNA target genes annotated in the given pathway.

### 2.3. Identification of Risk Genes and miRNAs Related to Breast Cancer Subtypes

For each breast cancer subtype, we identified significant differentially expressed genes/miRNAs by comparing the tumor with normal samples in each subtype. The unpaired Student’s *t*-test and fold-change methods were simultaneously used to evaluate differentially expressed genes/miRNAs. Then, the significance *p*-values from the *t*-test were calibrated by Benjamini-Hochberg multiple tests to obtain the false discovery rate (FDR) values. Finally, we applied *p* < 0.01 and $\left|\log_{2}\mathrm{FC}\right|$ > 2 as thresholds to identify differentially expressed genes/miRNAs. These significant differentially expressed genes/miRNAs were regarded as breast cancer subtype-associated genes, which were also defined by us as risk genes and miRNAs, respectively.

### 2.4. Mining Risk Pathways Associated with Breast Cancer Subtypes

In order to explore the roles of these risk genes and miRNAs in the occurrence and development of breast cancer, we performed them to conduct pathway enrichment analysis to dig out the pathways closely related to breast cancer. We identified pathways with significant enrichment results as risk pathways for each subtype based on risk genes and miRNAs. The cumulative hypergeometric test was used to calculate the significance of each pathway that enriched by risk genes and miRNAs. The formula of the cumulative hypergeometric test is as follows:

$$P=1-\overset{m}{\underset{k=0}{\sum }}\frac{\left(\begin{array}{c} n\\ k \end{array}\right)\left(\begin{array}{c} N-n\\ M-k \end{array}\right)}{\left(\begin{array}{c} N\\ M \end{array}\right)}$$

where N represents the number of background genes (all genome-wide genes), M is the number of a given pathway’s genes and miRNAs that are annotated in the N genes, n is total number of the risk genes and miRNAs of a given subtype of breast cancer, and m is the number of risk genes and miRNAs in the given pathway.

### 2.5. Establishing the Risk Pathways’ Crosstalk of Breast Cancer

In each breast cancer subtype, we calculated the crosstalk of each pair of risk pathways based on the correlation strength of genes and miRNAs between them according to previous studies [21]. The Pearson’s product moment correlation coefficient and unpaired Student’s *t*-test were performed to measure correlation strength for any two interrelated pathways. As for genes and miRNAs presenting both in pathway i and j, we reckoned their correlation strength only if they interact with other genes or miRNAs in the PPI network. Then, we used correlation strength to construct and assess risk pathways’ crosstalk. The formula of calculating correlation strength is as follows:

$$CS\left(i,j\right)=F\left(P\left(i\right),P\left(j\right)|Exp_{i},Exp_{j}\right)\;=-2*\left(log_{e}P\left(i\right)+log_{e}P\left(j\right)+log_{e}P\left(i,j\right)\right)$$

where i is the gene that is annotated in pathway a;j is the gene that is annotated in pathway $b;\;Exp_{i}$ and $Exp_{j}$ are the expression values of genes i and j in samples, respectively; $P\left(i\right)$ and $P\left(j\right)$ are the differential significance *p*-values of genes i and j calculated using the unpaired Student’s *t*-test, respectively; and P(i,j) is the significant *p*-value of expression correlation coefficient between a and b genes/miRNAs based on the Pearson’s product moment correlation coefficient.

The crosstalk of any pair of risk pathways was gained by adding up all the correlation strengths between them, and crosstalk of risk pathways i and j was developed based on formula as follows:

$$Crosstalk_{\left(a,b\right)}=\overset{n}{\underset{a}{\sum }}CS$$

where *n* presents the number of all gene–gene, gene–miRNA, and miRNA–miRNA interactions between any two pathways.

In order to strengthen the differences of risk pathways in different subtypes, we constructed specific dysfunctional crosstalk networks based on the specific crosstalk relationship in each subtype for subsequent calculation and research, which means that when a pair of crosstalk pathways only exist in a certain subtype, they will be selected to construct the subtype crosstalk network.

### 2.6. Evaluating the Impacts of Drugs on Crosstalk

We integrated the drug information from the DrugBank and SM2miR databases and screened them for Food and Drug Administration (FDA)-approved anticancer drugs that contain both target genes and target miRNAs, and a total of 36 anticancer drugs were screened. Research has shown that the crosstalk among the signaling pathways plays a key role in the occurrence and development of breast cancer. Thus, evaluating the impact of drugs on pathway crosstalk based on the expression of drug targets could help to optimize the treatment of various subtypes of breast cancer. From this standpoint, in order to assess the impacts of drug on dysfunction crosstalk network, for each drug, we first removed its target genes and miRNAs from the specific risk pathway crosstalk of a given subtype. Next, we recalculated the crosstalk to quantify the destructive effects of drugs on different subtypes. At the same time, a formula was designed and developed. The destructive score (*DS*) of drug to crosstalk was gained using the following formula:

$$DS\left(d\right)=\frac{\sum_{i}^{k}\left|1-\frac{Crosstalk_{d}}{Crosstalk}\right|}{k}$$

where $Crosstalk_{d}$ is the crosstalk after drug action, and k presents the number of all specific crosstalks in the subtype.

We determined the destructive score (*DS*) of all anticancer drugs to specific crosstalk networks in each subtype to assess the impacts of drugs on pathway crosstalk of the drugs. A higher *DS* score indicates the greater effects of the drug on crosstalk between risk pathways. In each subtype, we only screened anticancer drugs that could impact the crosstalk between dysregulated pathways (*DS* score greater than zero) as candidate drugs, and we ranked candidate drugs of each subtype by *DS* score from high to low in various subtypes of breast cancer. 

### 2.7. Survival Analysis

We performed survival analysis based on the targets of candidate drugs that were implicated in the specific pathway crosstalk of each subtypes of breast cancer to evaluate the effects for patient survival of candidate drugs. For a given drug, we extracted its target genes and miRNAs that target a specific crosstalk network as drug target signatures. Each candidate drug target signature was performed for survival analysis in patients of each subtype separately, and we used the K-mean clustering method to stratify patients into shorter survival time and longer survival time groups based on the level of these drug target molecules’ expression. In this project, we used 100 as the maximum number of iterations of k means algorithm, and randomly started k means algorithm 20 times to return the best result. Then Kaplan–Meier estimate method was used to evaluate the survival difference of these two classified groups in each subtype, respectively. Finally, the significance *p*-value of survival difference was estimated using the log-rank test.

## 3. Results

### 3.1. Identifying Breast Cancer Subtype-Associated Risk Pathways

**Figure 2:**
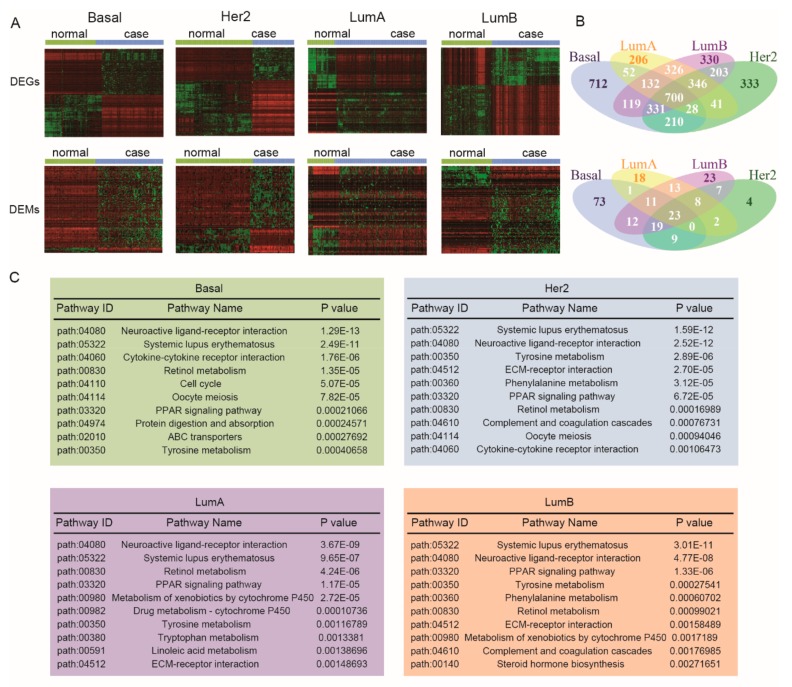
Global view of risk genes and miRNAs in each subtype of breast cancer. (**A**) Heat maps show risk genes and miRNAs in four breast cancer subtypes. Unsupervised hierarchical clustering analysis is used, which divided genes and miRNAs into two clusters, the lower and higher expression values are represented by green and the red colors, respectively. (**B**) Venn plots of risk genes and miRNAs associated with breast cancer subtypes separately. (**C**) Results of top 10 pathways with significant enrichment result of each subtype. Note: Basal, basal-like subtype; Her2, her2-enriched subtype; LumA, luminal A subtype; LumB, luminal B subtype.

We identified the risk miRNAs and genes by comparing tumor samples in each subtype with normal controls, respectively. The differentially expressed genes and miRNAs were detected using *t*-test and fold-change methods, and then multiple testing correction by the Benjamini–Hochberg procedure was used. Genes/miRNAs with adjusted *p*-values < 0.01and |log_2 FC| > 2 were identified as differential expression (risk genes/miRNAs). In total, we obtained 4096 risk genes (2284 from basal-like subtype, 2192 from her2-enriched subtype, 1831 from luminal A subtype, and 2487 from luminal B subtype) and 223 risk miRNAs (148 from basal-like subtype, 72 from her2-enriched subtype, 76 from luminal A subtype, and 116 from luminal B subtype). Unsupervised hierarchical clustering analysis was performed to observe discrepancy of the expression of risk genes and miRNAs between case samples and normal samples, as shown in Figure 2A. We also performed the degree of overlap of risk genes and miRNAs between subtypes, displayed in Figure 2B. These results indicate that genes and miRNAs exhibit widespread expression disorder in the various breast cancer subtypes. 

Breast cancer is affected by multiple factors and pathways. In order to veritably and accurately reflect the changes of the pathways of breast cancer, we used the methods that we developed previously to reconstruct all biological pathways among KEGG, and miRNAs were added into the signaling pathway to form a more abundant signaling pathway. To discover the biological function of these risk genes and miRNAs, we used pathway enrichment analysis to identify risk pathways in each subtype. A pathway is identified as a risk pathway only if risk genes and miRNAs are enriched in it under the significance level *p* < 0.05. In total, there were 32 risk pathways in basal-like subtype, 29 risk pathways in her2-enriched subtype, 21 risk pathways in luminal A subtype, and 26 risk pathways in luminal B subtype. We show the top ten pathways of each breast cancer subtype in Figure 2C. We found that some risk pathways such as the Chemokine signaling pathway, ECM–receptor interaction, the PPAR signaling pathway, and Tyrosine metabolism were simultaneously identified in different breast cancer subtypes. Furthermore, we found some subtype-specific risk pathways in each subtype of breast cancer. Amoebiasis, drug metabolism–other enzymes, fatty acid metabolism, the p53 signaling pathway, and salivary secretion were found in basal-like, cell adhesion molecules (CAMs) in her2-enriched, histidine metabolism in Luminal A, and glycerolipid metabolism and TGF-beta signaling pathway in Luminal B subtypes. These subtype-specific risk pathways may be one of the reasons that resulted in distinct molecular mechanisms and clinical outcomes of breast cancer subtypes.

### 3.2. Constructing Risk Pathway Crosstalk Networks for Various Subtypes of Breast Cancer

The occurrence of breast cancer is complex and there is crosstalk between different functional biological pathways in the process of cancer development. Thus, it is necessary to dissect the crosstalk of dysfunctional pathways related to breast cancer. To elucidate the molecular mechanism of various breast cancer subtypes, we analyzed the crosstalk between dysfunctional pathways that are related to breast cancer. In our study, the risk pathway crosstalk networks for each breast cancer subtype were constructed. The quantification of crosstalk was conducted by calculating both the correlation strength and the dysfunction degree of genes and miRNAs in any two risk pathways of each breast cancer subtype, and the expression correlation coefficient between genes and miRNAs and the unpaired Student’s t-test of genes and miRNAs were used for assessment of crosstalk.

**Figure 3:**
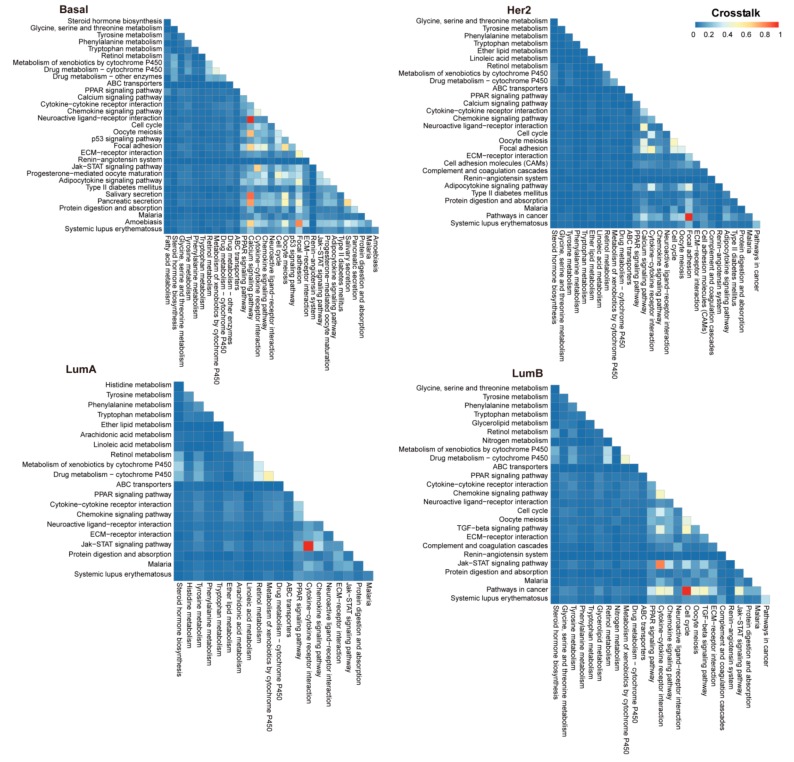
The crosstalk for each two interrelated risk pathways in breast cancer subtypes. Heat maps of crosstalk between risk pathways for comparing the heterogeneity of crosstalk across different subtypes of breast cancer. The color of the box represents the crosstalk between the two pathways, the lower and higher crosstalk are represented by blue and the red colors, respectively.

Our results showed that there were crosstalks with significant differences in the extent of crosstalk between risk pathways in each subtype (Figure 3). For example, ‘calcium signaling pathway’ and ‘focal adhesion’ have more crosstalk relationships with other pathways in basal-like subtype. ‘Pathways in cancer’ and ‘focal adhesion’ crosstalk more with other pathways in her2-enriched subtype. In luminal A subtype, ‘Jak−STAT signaling pathway’ has the greatest crosstalk with ‘cytokine−cytokine receptor interaction’. In luminal B subtype, ‘pathways in cancer’ and ‘cytokine−cytokine receptor interaction’ possess larger crosstalk values with other pathways.

**Figure 4:**
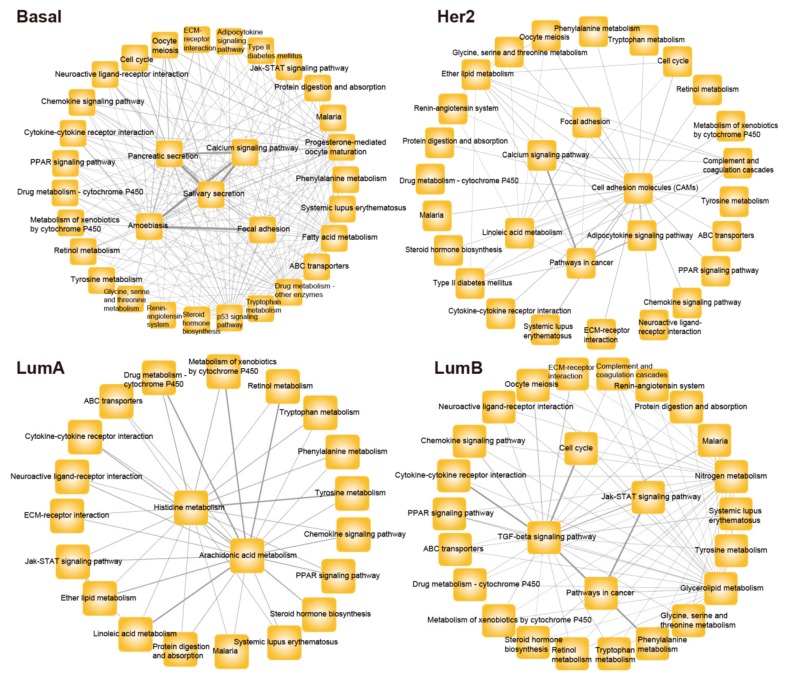
The specific crosstalk network of each breast cancer subtype. The yellow rectangle represents the pathways of the specific crosstalk network. The thickness of edges represents the intensity of crosstalk between pathways; the larger the crosstalk value, the thicker the edge.

Moreover, we found some subtype-specific crosstalk of pathways in breast cancer. We extracted the specific crosstalk risk pathways of each subtype and used them to construct the specific crosstalk network of the risk pathway in four subtypes (Figure 4). There are 197 specific crosstalk relationships in basal-like, 56 specific crosstalk relationships in her2-enriched, 41 specific crosstalk relationships in luminal A, and 74 specific crosstalk relationships in luminal B subtypes. The above results indicate that these subtype-specific crosstalks of risk pathways may be one of the molecular mechanisms that lead to distinct clinical outcomes of breast cancer patients, which will help us to understand the discrepancy between subtypes and points a new way to optimize the treatment of breast cancer patients.

### 3.3. Screening Candidate Therapeutic Drugs for Each Subtype of Breast Cancer Based on DS Score

Previous experimental studies have demonstrated that cancer cells could adapt signaling pathway circuits under drug treatment by establishing alternative signaling routes through crosstalk [32,33]. Based on this point of view, we developed an evaluation method to optimize the therapeutic drugs for each subtype of breast cancer by assessing the impact of drugs on crosstalk among risk pathways. The drug targets of each drug were removed from risk pathways and we reconstructed crosstalk networks targeted by drugs to evaluate the perturbance effects of those drugs. Next, we recalculated the crosstalk to measure the perturbance effects of drugs on different subtypes and optimize the drug use for each subtype of breast cancer. We obtained 36 anticancer drugs that target both genes and miRNAs, and the results of evaluation of anticancer drugs are shown in Table 1. We only screened anticancer drugs of each subtype with a *DS* score greater than zero as candidate drugs, and ranked candidate drugs of each subtype by *DS* score from high to low. A higher DS score indicates the greater effects of the drug on crosstalk between risk pathways. In total, there are 33 drugs in basal-like, 32 drugs in her2-enriched, 22 drugs in luminal A, and 30 drugs in luminal B subtypes. 

### 3.4. Dissecting the Effects of Candidate Therapeutic Drugs for Patient Survival in Each Subtype of Breast Cancer

**Figure 5:**
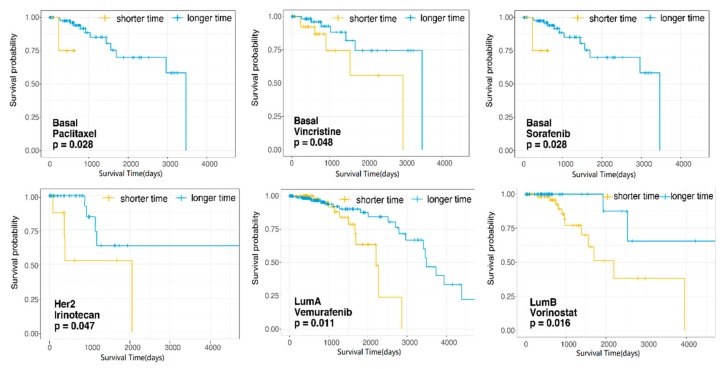
Kaplan-Meier survival curves of patients at shorter survival time group or longer survival time group stratified by drug target signatures of candidate drugs of each breast cancer subtype.

A drug could specifically interact with a target molecule to modulate a physiological process and further impact the progression of a disease [34]. In order to further screen drugs for breast cancer patients, we got the patients’ clinical survival information in each breast cancer subtype. For each candidate therapeutic drug that was screened based on DS score in different subtypes, we evaluated the drug target signature’s influence on patient survival. Patients from each subtype of breast cancer were divided into two groups (shorter survival time group and longer survival time group) based on the expression of drug target signatures. As shown in Figure 5, we found that there were, in total, six candidate therapeutic drugs screened based on DS score (DS score greater than zero) that significantly correlated with overall survival (OS) in the different subtypes of breast cancer patients. Paclitaxel, Vincristine, and Sorafenib in basal-like, Irinotecan in her2-enriched, Vemurafenib in luminal A, and Vorinostat in luminal B subtypes. These six dugs not only impacted the crosstalk of risk pathways, but they also had an effect on the patients’ survival in their corresponding subtypes. This indicates that they may be more suitable treatment candidates for the corresponding subtypes of breast cancer. More details, according to drug target signatures of Paclitaxel and Sorafenib in the basal-like subtype, these 97 patients were divided into a shorter survival group (*n =* 5) and a longer survival group (*n =* 92), respectively. Vincristine drug target signatures divided 97 patients in the basal-like subtype into a shorter survival group (*n =* 63) and a longer survival group (*n =* 55). The 47 patients in the her2-enriched subtype were separated into a shorter survival group (*n =* 10) and a longer survival group (*n =* 37) by Irinotecan drug target signatures. Based on the drug target signatures of Vemurafenib in luminal A subtype, the 287 patients (survival information was missing in four patients) were stratified into a shorter survival group (*n =* 78) and a longer survival group (*n =* 209), and Vorinostat drug target signatures stratified 118 luminal B subtype patients into a shorter survival group (*n =* 63) and a longer survival group (*n =* 55). Here, drugs’ signatures stratified the patients into two groups in a statistically significant manner and their expression direction were not considered.

### 3.5. Dissecting the Mechanism of Candidate Drugs for Each Subtype

**Figure 6:**
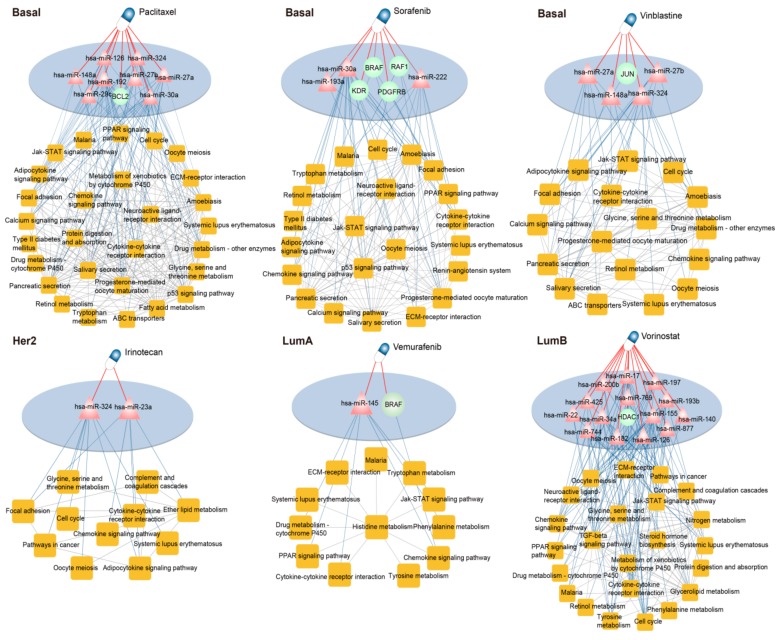
The mechanism of optimal therapeutic drugs in each subtype of breast cancer. Sorafenib, Paclitaxel, and Vincristine were applicable for the basal-like subtype treatment, Irinotecan was optimum for the her2-enriched subtype treatment, Vemurafenib was suitable for the luminal A subtype treatment, and Vorinostat was applied to the luminal B subtype treatment.

In our drugs’ optimization results, Paclitaxel, Sorafenib, and Vincristine were found to have potential therapy effect in the basal-like subtype of breast cancer. Consistent with clinical findings, Paclitaxel and Vincristine were the optimal adjuvant therapy for triple-negative breast cancer [35,36]. Sorafenib is a multiple targeted agent which can inhibit tumor cell proliferation and angiogenesis by inhibiting the activation of multiple different kinases [37], and our results indicate that Sorafenib plays a therapeutic role in the basal-like subtype of breast cancer mainly through affecting specific risk pathway crosstalk mediated by hsa-miR-30a, hsa-miR-222, and hsa-miR-193a. Some studies have confirmed that hsa-miR-30a, hsa-miR-222, and hsa-miR-193a play key roles in breast cancer [38,39,40]. Irinotecan, an antitumor enzyme inhibitor mainly used for the treatment of colorectal cancer [41], is suitable for the her2-enriched subtype, which mediates the specific crosstalk among the risk pathways of the her2-enriched subtype through regulating hsa-miR-23a and hsa-miR-324. In accordance with the result of WT Kuo and Eissa [42,43], hsa-miR-324 and hsa-miR-23a have distinct biological functions in breast cancer. Vemurafenib has long been approved for the treatment of metastatic melanoma with BRAF mutation [44], and our results showed that this drug had a damaging effect on the specific crosstalk of risk pathway of the luminal A subtype through action on hsa-miR-145. Just as some researches have shown that miR-145 is a potential cancer biomarker and serves as a novel target for cancer therapy, including breast cancer [45]. Vorinostat as an anticancer agent that inhibits histone deacetylases, approved for cutaneous T-cell lymphoma [46], and plays a key role in the epigenetic regulation of gene expression. Vorinostat could act on the specific risk pathways crosstalk of the luminal B subtype via 14 miRNAs (Figure 6), which have been found to play important roles in the occurrence and development of breast cancer, such as hsa-miR-155, hsa-miR-34a, hsa-miR-17, hsa-miR-22, and hsa-miR-140 [47,48,49,50,51].

## 4. Discussion

Breast cancer is a complex disease with high heterogeneity in terms of the underlying molecular alterations, the cellular composition of tumors, and even the clinical outcomes. Different subtypes exhibit distinct biological behavior, prognosis, and usually different responses to drug treatment [52], yet identifing applicable drugs for each subtype still largely remains limited. Therefore, it is urgently needed to develop a systematic pipeline to identify medications for different subtypes of breast cancer.

The occurrence and development of tumors is a complex process involving many steps, links, and factors. It is mostly the action of a single molecule (gene or miRNA) that leads to poor therapeutic effect among many chemotherapeutic regimens [53]. In recent years, many researches have revealed that the occurrence of tumors is closely related to the abnormality of biological pathways, and crosstalk of abnormal pathways is one of the prime reasons for the poor outcomes of tumor treatment [54]. Studies have shown that regulatory molecules such as non-coding RNA participate in the anomaly of biological pathways through the regulation of genes, adding to the difficulty of cancer treatment [55]. In order to actually reflect the intricate crosstalk of pathways, we have developed a new method based on biological pathways—that is, reconstruction of biological pathways which include both genes and miRNAs. We have also identified the optimal drugs by quantifying the effect of candidate drugs on miRNA-mediated crosstalk of pathways. We have successfully identified the specific crosstalk of pathways in each subtype of breast cancer and revealed their pathogenesis respectively by applying this method. Moreover, we also screened applicable drugs for each subtype of breast cancer. We successfully screened the most suitable drugs for each subtype of breast cancer, including Paclitaxel and Vincristine, which are breast cancer treatment drugs in clinical application. On the basis of the original application, we accurately identified their applications in each subtype, such that Paclitaxel and Vincristine were best for basal-like, Irinotecan was suitable for her2-enriched, and Vorinostat was the optimal drug for luminal B subtypes. We also identified other anticancer drugs application in each subtypes of breast cancer. The results show that our approach could help doctors to further improve treatment strategies with the current menu of chemotherapy options.

Currently, several methods have been proposed to optimize drugs for human cancers. For example, Lamb et al. provided a computational method to connect diseases and their potential therapeutic small molecules based on gene expression profiles form disease and cultured human cells treated with bioactive small molecules respectively [56]. Gottlieb et al. predicted novel drug indications based on multiple drug–drug and disease–disease similarity measures [57]. Furthermore, Malas et al. prioritized drugs using the semantic information between drug and disease concepts [58]. Comparing with these methods, our study has some unique features. First, we considered the role of non-coding RNAs in our approach. Second, our study optimized anticancer drugs by measuring their effects for mediating the crosstalk between risk pathways, which was an important molecular mechanism in the initiation and progression of human cancers. Finally, we optimized candidate drugs for different breast cancer subtypes, which may further promote the precise use of drugs for human cancer.

There are also several limitations in our study. First of all, drugs targeting miRNAs for therapeutic purposes are limited, and there are many drugs without miRNA targets. Secondly, miRNAs affected by the drugs are required for further study. We believe that more and more drugs that regulate miRNAs and drug-regulated miRNAs will be discovered with the development of in-depth study on the interaction of drugs and miRNAs, and our method can identify the optimal therapeutic agent for complex diseases more accurately and comprehensively. In summary, the results in this study highlight that dissecting subtype-specific risk pathway crosstalk could provide novel insights into the underlying molecular mechanisms and thus promote the drug discovery for various breast cancer subtype. Moreover, we focused on breast cancer in this study, but the method proposed here could also be applied to many other complex diseases, as pathway crosstalk is widespread in biological systems and the dysregulation of which play a critical role in the occurrence of disease.

## Figures and Tables

**Table 1:** Screened candidate drugs for various subtypes of breast cancer based on *DS* score.

*DS* Score Ranking	Basal	Her2	LumA	LumB
1	5-Fluorouracil	Arsenic trioxide	Arsenic trioxide	Arsenic trioxide
2	Arsenic trioxide	Adriamycin	5-Fluorouracil	Adriamycin
3	Tamoxifen	5-Fluorouracil	Adriamycin	5-Fluorouracil
4	Trastuzumab	Trastuzumab	Trastuzumab	Trastuzumab
5	Etoposide	Paclitaxel	Etoposide	Etoposide
6	Cisplatin	Temozolomide	Tamoxifen	Cisplatin
7	Paclitaxel	Etoposide	Vorinostat	Topotecan
8	Vorinostat	Gemcitabine	Bicalutamide	Irinotecan
9	Gemcitabine	Everolimus	Cisplatin	Paclitaxel
10	Adriamycin	Sunitinib	Vemurafenib	Tamoxifen
11	Temozolomide	Tamoxifen	Medroxyprogesterone acetate	Vemurafenib
12	Cyclophosphamide	Vorinostat	Gemcitabine	Gemcitabine
13	Bicalutamide	Cisplatin	Temozolomide	Sunitinib
14	Sunitinib	Sorafenib	Everolimus	Vorinostat
15	Vemurafenib	Cyclophosphamide	Sunitinib	Temozolomide
16	Medroxyprogesterone acetate	Goserelin	Paclitaxel	Everolimus
17	Everolimus	Vemurafenib	Oxaliplatin	Lenalidomide
18	Vinblastine	Bicalutamide	Cyclophosphamide	Cyclophosphamide
19	Lenalidomide	Vinblastine	Sorafenib	Bicalutamide
20	Oxaliplatin	Lenalidomide	Irinotecan	Goserelin
21	Sorafenib	Imatinib mesylate	Topotecan	Rapamycin
22	Goserelin	Bortezomib	Lenalidomide	Oxaliplatin
23	Irinotecan	Oxaliplatin		Vinblastine
24	Mitoxantrone	Medroxyprogesterone acetate		Sorafenib
25	Topotecan	Melphalan		Vincristine
26	Imatinib mesylate	Gefitinib		Medroxyprogesterone acetate
27	Vincristine	Rapamycin		Bortezomib
28	Gefitinib	Vincristine		Imatinib mesylate
29	Docetaxel	Irinotecan		Mitoxantrone
30	Bortezomib	Topotecan		Melphalan
31	Melphalan	Mitoxantrone		
32	Rapamycin	Docetaxel		
33	Epirubicin			

## Supplementary Materials

The following are available online at https://www.mdpi.com/2073-4425/10/9/657/s1, Table S1: Detailed information of the 36 anticancer drugs and their targets.
